# Concentration-dependent effects of effusol and juncusol from *Juncus compressus* on seedling development of *Arabidopsis thaliana*

**DOI:** 10.1038/s41598-022-18063-5

**Published:** 2022-08-16

**Authors:** László Bakacsy, Lilla Sípos, Anita Barta, Dóra Stefkó, Andrea Vasas, Ágnes Szepesi

**Affiliations:** 1 Department of Plant Biology, Institute of Biology, Faculty of Science and Informatics, University of Szeged, Közép Fasor 52., Szeged, 6726 Hungary; 2 Department of Pharmacognosy, Faculty of Pharmacy, University of Szeged, Eötvös u. 6, Szeged, 6720 Hungary

**Keywords:** Plant sciences, Plant development, Plant physiology, Plant stress responses

## Abstract

*Juncus* species are valuable sources of phenanthrene compounds that have been used in traditional Chinese medicine for thousands of years. Effusol and juncusol are the most investigated compounds reported to have antimicrobial and anticancer effects; however, to date, their effects on higher plants have not been investigated. In this study, we examined the effects of effusol and juncusol on the growth and other biochemical parameters of the dicot model plant *Arabidopsis thaliana* in a concentration-dependent manner with a focus on polyamine metabolism. Phenanthrene induced toxic effects on plant growth and development, while effusol and juncusol induced higher biomass and maintained antioxidant defence mechanisms associated with reduced polyamine degradation. Taken together, our results suggest that these compounds could be good candidates for new biopesticide or biostimulant plant growth regulators in the future.

## Introduction

Plants have a number of specific metabolites that are important not only for human healing but also for more sustainable agriculture. The research and use of these natural active ingredients is thus receiving increasing attention. Pests and diseases are a constant threat to agricultural production, and climate change will further increase their occurrence, prevalence and impact^1^. The use of synthetic pesticides and fertilizers should therefore be reduced or avoided whenever possible^1^. EU policy is directed towards significant reductions in pesticide use in the short to medium term^2^. There is growing demand to replace chemical pesticides or fertilizers with alternatives such as natural growth-promoting compounds to reach sustainable food production goals and protect the environment. Conventional pesticide use has become controversial in many countries, and some European Union (EU) Member States have adopted policies for risk reduction following Directive 2009/128/EC, the sustainable use of pesticides^3,4^. Control of pathogens by means of plant‐derived plant protection products can be an effective, sustainable and environmentally friendly method for pest management in integrated pest management (IPM) and organic farming systems^5^.

Rushes (*Juncus* sp.) are good candidate plant species for finding new bioactive compounds. They are cosmopolitan, and their representatives can be found on every continent. These plants can occur in a variety of habitats, with some species found in wet swamps to dry, nutrient-poor soils. These plants contain a number of valuable secondary metabolites^6^ that have contributed to the wide application of rushes to traditional Chinese medicine (TCM) as herbs to treat various human diseases for thousands of years^7^. Most of the studies used extracts from rushes as a mixture of these compounds without any precise analytical purification of each metabolite^1^. These compounds can be used for biocontrol and pest control^1,8,9^. Currently, the development of analytical technologies has contributed to the discovery of novel compounds that may be new promising metabolites with many applications.

These secondary metabolites have diverse biological activities. The main specific metabolites of *Juncus* species belong to the group of phenanthrenes^10,11^. Natural phenanthrenes, called phenanthrenoids, are the most common of these metabolites and often follow evolutionary patterns and taxonomic divisions^12^. Phenanthrenoids, as a class of aromatic metabolites, belong to the stilbenoids^10,13^ and act as phytoalexins after pathogen infections in plants^14^. These phenanthrenes are naturally synthesized from the stilbenoid pathway in plants^15^, and can provide plants with protection against biotic and abiotic stress^16^. Phenanthrene (Phe) is the basic structure of phenanthrene-like compounds. However, it is important to note that Phe is a hazardous chemical compound threatening the environment^17^. In addition to much data on its toxicity to animals, the effects of Phe have been widely examined in many studies that demonstrated its toxicity to higher plants. *Arabidopsis thaliana* is one of the most widely used model plant species in bioactivity studies and is used to examine the effects of different natural compounds^18^. Effusol and juncusol are the two most abundant and studied metabolites of rushes with a Phe structure^19,20^. The properties of these metabolites have been studied in many organisms, including their antialgal activity on green algae^21^, antiproliferative activity on the HeLa human cervical cancer cell line^22^, and antiviral and antibacterial effects on different strains^23^. Some bacteria, e.g., *Achromobacter* sp., *Burkholderia* sp., *Mycobacterium* sp., *Rhodococcus* sp. and *Pseudomonas aeruginosa,* are able to degrade Phe through the salicylate pathway^24–26^. The initial step of the degradation is a dioxygenation mainly at C-5,6 on the C ring. Thereafter, salicylic acid can be formed from 1-hydroxy-2-naphthoic acid through the intermediate 1,2-dihydroxynaphthalene. Salicylic acid can enhance the accumulation of glutathione, thus contributing to detoxification and the antioxidant system, which further enhances Phe degradation and Phe stress tolerance in plants^24,25^.

Polyamines (PAs) are essential polycationic compounds involved in growth, development and stress tolerance in plants. Burritt^27^ demonstrated that PAs can have protective roles in Phe-induced damage in the aquatic liverwort *Riccia fluitans* L. The most important PAs could act as stress markers when found in different forms, such as free, membrane bound or conjugated^28,29^. Under stress conditions, enhanced levels of free PAs, including the most commonly investigated diamine putrescine (Put), triamine spermidine (Spd) and tetramine spermine (Spm), are reported to contribute to stress tolerance in plants^30,31^. Degradation of these free PAs is catalysed by two different enzymes, the copper-containing diamine oxidase (DAO) and the flavine-containing polyamine oxidase (PAO); both contribute to the fine tuning of PA metabolism^32,33^.

However, the effects of effusol and juncusol have not been investigated in higher plants, so we aimed to determine the physiological responses of *Arabidopsis thaliana* to these phenanthrenes. In our experiments, Phe was used as a control as it shares the basic structure of both metabolites. We hypothesized that both compounds would show more beneficial effects than Phe in this higher plant, so we selected three concentrations for the experiments (0, 0.1, 0.5, and 1 mM). Growth parameters (biomass, leaf area, and primary root length), photosynthetic pigments, some physiological parameters [protein level, hydrogen peroxide (H_2_O_2_) content, superoxide dismutase (SOD), catalase (CAT), and guaiacol peroxidase (POD)], and the stress markers PAs and their degrading enzyme activities were measured to evaluate the effects of these natural phenanthrene-like compounds in *Arabidopsis thaliana* seedlings.

## Materials and methods

### Plant material, growth conditions and treatments

**Figure 1:**
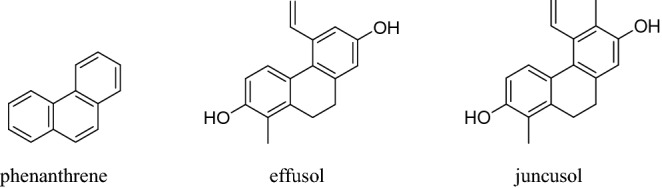
Structures of phenanthrene, effusol and juncusol.

The *Arabidopsis thaliana* Columbia-0 ecotype was used for the experiments. Seeds were provided by Edit Horváth (Department of Plant Biology, University of Szeged). Seeds were surface sterilized with 70% (v/v) ethanol and 20% bleach (v/v) solution. After washing with distilled water, the seeds were stored at 4 °C for 1 day. Seedlings were grown in the greenhouse of the Department of Plant Biology, University of Szeged. Seeds were planted on 0.5 × Murashige and Skoog agar (0.8%) medium^34^ with the addition of 0.5% sucrose (w/v) (pH adjusted to 5.5 with NaOH) in plastic round Petri dishes (90 × 17 mm) with five seeds per Petri dish in a single line. The experimental setup was designed as described by Marik et al.^18^, and Petri dishes were positioned vertically. Plants were grown for 5 days in a controlled environment under 200 µmol m^−2^ s^−1^ photon flux density (F36W/GRO lamps, OSRAM SYLVANIA, Danvers, MA, USA) with a 12/12-h light/dark period, day/night temperatures of 24/22 °C and a relative humidity of 55–60%^31^. Five-millimetre holes were bored in agar with a sterile cork borer 0.5 cm from the root tips of 5-day-old *Arabidopsis* seedlings. Treatments were conducted by filling the hole with 10 μL of three different concentrations (0.1, 0.5 and 1 mM) of Phe, effusol and juncusol dissolved in methanol (Fig. 1). Effusol and juncusol were provided by Andrea Vasas (Faculty of Pharmacy, University of Szeged), and Phe was purchased from Merck Millipore (Darmstadt, Germany).

For control plant treatments, methanol was pipetted into the holes. Sampling of the whole seedling was conducted after 1 week of treatment.

### Growth parameters of *Arabidopsis thaliana*

The biomass of plants grown with the treatment compounds was measured by an analytical scale (Adam Equipment NBL2541, Milton Keynes, United Kingdom), and the samples were kept at − 20 °C for biochemical analyses. The growth of *A. thaliana* seedlings was monitored by image capture (Canon EOS 700D, Canon, United Kingdom). Petri dishes were photographed without a lid from above using a homogenous surface. Images from the Petri dishes were saved in jpeg format, and then leaf areas and root lengths were analysed by ImageJ software ver. 1.52a (National Institute of Mental Health, Bethesda, Maryland, USA) (http://imagej.nih.gov/ij)^35^. The experiments were repeated three times.

### Determination of photosynthetic pigment contents from *Arabidopsis* seedlings

The fresh weights of the whole plants from each plate were measured, and photosynthetic pigments were quantified as described by Faragó et al.^36^. Fresh tissue was ground with ethanol, and the homogenate was centrifuged (Eppendorf centrifuge 5424R, Eppendorf GMBH, Germany) at 12 000 rpm and 4 °C for 10 min. The optical densities of the supernatants were detected by a plate reader (Synergy HTX plate reader, BioTek Instruments, Winooski, VT, USA) at 664, 648 and 470 nm. Calculations for chlorophyll-a, chlorophyll-b and carotenoids were described by Faragó et al.^36^, and pigment contents were normalized to 1 g fresh weight.

### Total soluble protein content determination

Soluble proteins were extracted from 100 mg of frozen seedling samples. Homogenization was performed by the Bradford method^37^. Samples were ground in ice-cold phosphate buffer (KH_2_PO_4_ and Na_2_HPO_4_, 50 mM, pH 7.0) and centrifuged in an Eppendorf centrifuge (Eppendorf 5424R, Eppendorf GMBH, Germany) for 10 min at 4 °C. The supernatant was used to measure the total soluble protein content by a plate reader at 595 nm (Synergy HTX plate reader, BioTek Instruments, Winooski, VT, USA).

### Hydrogen peroxide (H_2_O_2_) content measurements

The H_2_O_2_ levels of *Arabidopsis* seedlings were measured as described by Horváth et al.^34^. One hundred milligrams of sample was homogenized in 0.5 mL of ice-cold 0.1% TCA. After centrifugation, 0.25 mL of the supernatant was diluted with 0.25 mL of 50 mM potassium phosphate buffer (pH 7.0) and 0.5 mL of 1 M potassium iodide (KI) (in 50 mM potassium phosphate buffer, pH 7.0). The reaction was started with the addition of KI, and the sample was incubated for 10 min at 25 °C. The absorbance values were recorded by a spectrophotometer (KONTRON, Milano, Italy) at 390 nm. A standard curve was prepared using the H_2_O_2_ standard. The results were expressed as μmol H_2_O_2_ g^−1^ FW.

### Determination of antioxidant enzyme activities

Enzyme extracts were prepared as described by Horváth et al.^34^. SOD (EC 1.15.1.1) activity measurement was based on the ability of the enzyme to inhibit the photochemical reduction of p-nitro-blue tetrazolium chloride (Sigma-Aldrich) in the presence of riboflavin in the light. One enzyme unit (U) of SOD represents the amount of enzyme causing a 50% inhibition of p-nitro-blue tetrazolium chloride reduction. The enzyme activity was calculated as U g^–1^ fresh weight. CAT (EC 1.11.1.6) activity was determined by spectrophotometer based on the decomposition of H_2_O_2,_ and this decrease was measured based on the absorbance at 240 nm. One U was determined as the amount of H_2_O_2_ (in µmol) decomposed in 1 min during this reaction. During POD (EC 1.11.1.7) activity determination, the increase in absorbance was detected at 470 nm as the oxidation of guaiacol (molar extinction coefficient, ε470 = 26.6 mM^–1^ cm^–1^). The enzyme amount that could produce 1 µmol min^–1^ of oxidized guaiacol was determined to be 1 U.

### Free polyamine quantification

The three most important PAs are Put, Spd and Spm. Free PA contents were determined as described by Szepesi et al.^31^. In brief, 200 mg of seedlings was homogenized in 5% perchloric acid. After centrifugation, the supernatant was neutralized with 2 N NaOH, and then the PAs were derivatized with 10 µl of benzoyl chloride. Benzoyl-polyamines were dissolved in diethyl ether, evaporated and then separated by HPLC (JASCO, Tokyo, Japan). The applied standards were Put, Spd, and Spm in the form of hydrochlorides purchased from Merck Millipore (Darmstadt, Germany). Based on peak areas, the results were expressed in µmol g^−1^ fresh weight^−1^.

### Polyamine catabolic enzyme activity assays

Diamine oxidase (DAO, EC 1.4.3.6) and polyamine oxidase (PAO, EC 1.4.3.4) activities were estimated spectrophotometrically as described by Moschou et al.^38^ with some modifications. Two hundred milligrams of seedling tissue was homogenized in liquid N_2,_ and 0.6 mL extraction buffer was added to each sample. The extraction buffer contained 0.2 M TRIS (hydroxymethyl) aminomethane (pH 8.0), 10% glycerol, 0.25% Triton X-100, 0.5 mM phenylmethanesulfonyl fluoride (PMSF), and 0.01 mM leupeptin in 100 mM potassium phosphate buffer (pH 6.6). The homogenates were left on ice for 20 min and centrifuged for 10 min at 7000*g* at 4 °C. Then, 150 μL of the tissue extract was combined with 0.6 mL of 100 mM potassium phosphate buffer (pH 6.6), and the reaction was started by adding 22.5 μL of 2-aminobenzaldehyde (10 mg mL^−1^) and 1 M Put for DAO or 1 M Spd for PAO activity measurements. After incubation of the reaction mixture for 1.5 h at 37 °C, the reaction was stopped by adding 50 μL of 20% (w/v) trichloroacetic acid (TCA). The absorbance was determined at 430 nm by a plate reader (Synergy HTX plate reader, BioTek Instruments, Winooski, VT, USA). The enzyme activity was expressed as specific activity (U g^−1^ FW), where one unit (U) represents the amount of enzyme catalysing the formation of 1 μM Δ1-pyrroline min^−1^.

### Statistical analysis

The data presented are the mean values from at least three independent experiments. Statistical analysis was carried out with GraphPad Prism version 8.0.1.244 for Windows (GraphPad Software, La Jolla, CA, USA). Statistically significant differences were analysed by ANOVA followed by Tukey’s post-hoc test. The results were considered significant at P < 0.05, as indicated by different lowercase letters.

### Legal statement

All experiments and sample collection were performed in accordance with the relevant institutional, national, and international guidelines and legislation. We have also obtained corresponding permission to collect *Arabidopsis thaliana* seeds and plant material.

## Results

### Growth responses to Phe, effusol and juncusol treatments of *Arabidopsis thaliana* seedlings

**Figure 2:**
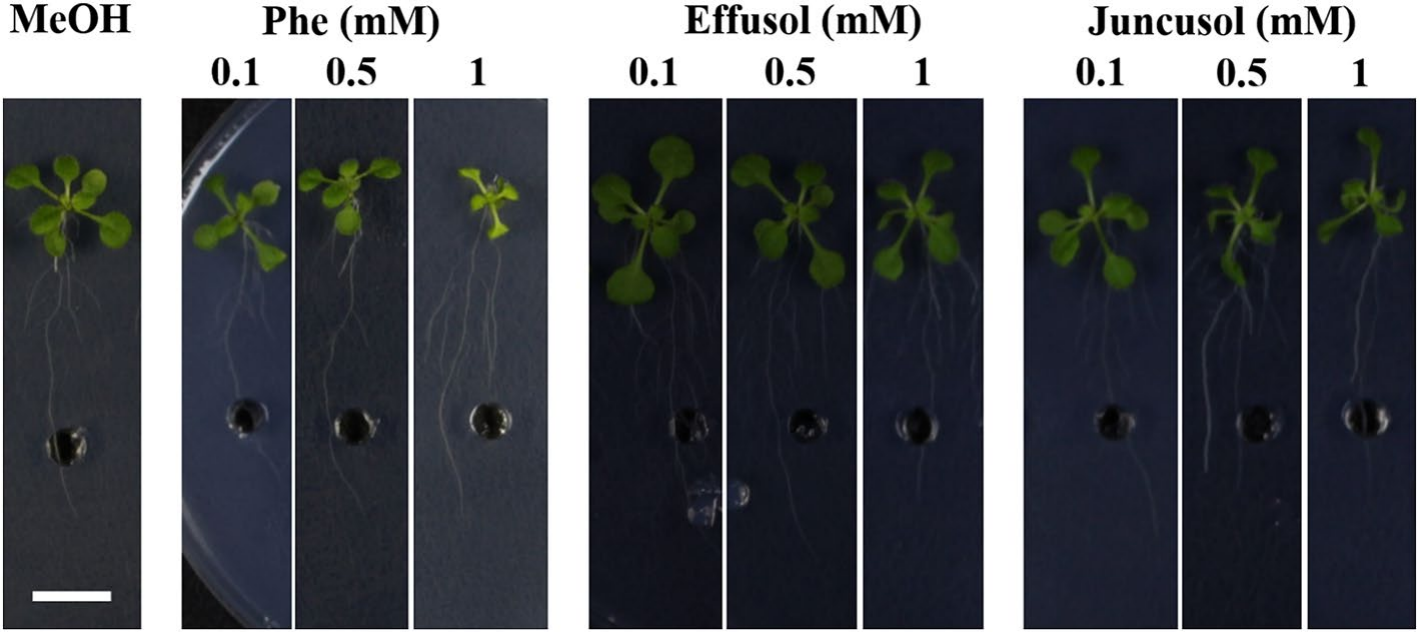
Representative images of *Arabidopsis thaliana* seedlings treated with phenanthrene (Phe), effusol and juncusol at different concentrations compared to methanol (MeOH). The applied concentrations were 0.1, 0.5 and 1 mM and were supplied via a hole in the agar plate. Scale bar 1 cm.

**Figure 3:**
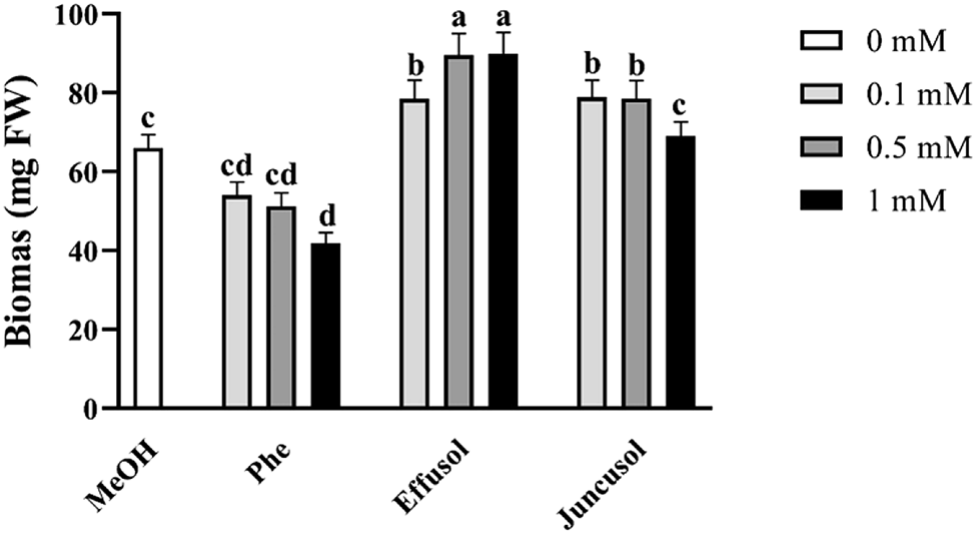
Biomass of seedlings treated with Phe, effusol and juncusol at different concentrations compared to MeOH. Data are presented as the mean ± SE (n = 30). Different letters indicate significant differences between the values of the treatments (P < 0.05) analysed by Tukey’s post-hoc test.

In this study, we aimed to investigate the biomass production of *A. thaliana* seedlings treated with Phe, effusol and juncusol. Previous studies provided evidence that Phe could drastically reduce plant growth. Phe treatment significantly decreased the biomass and leaf area of seedlings. Different trends could be seen for effusol and juncusol: effusol treatment increased plant biomass at all concentrations used, while juncusol increased plant biomass only at 0.1 and 0.5 mM compared to the methanol control (Figs. 2, 3).

**Figure 4:**
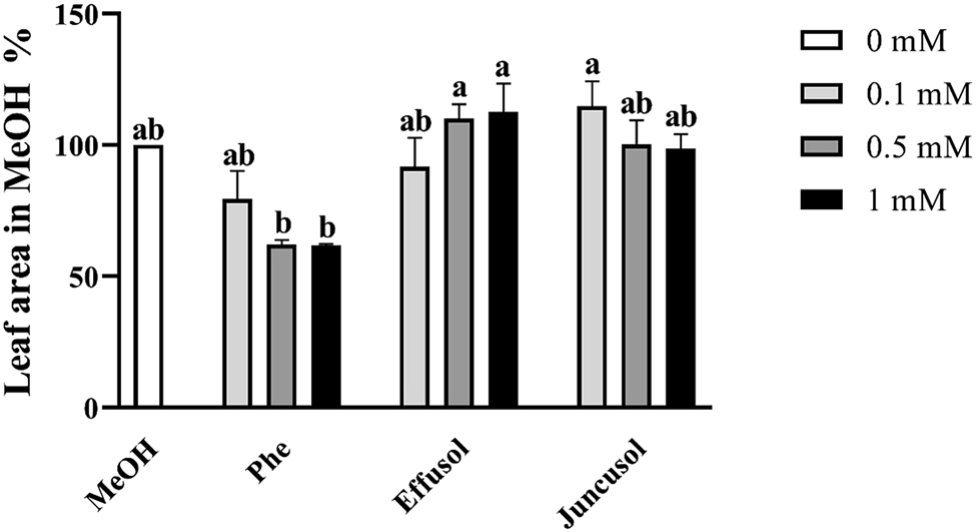
Leaf area of seedlings treated with Phe, effusol and juncusol at different concentrations compared to MeOH. Data are presented as the mean ± SE (n = 30). Different letters indicate significant differences between the values of the treatments (P < 0.05) analysed by Tukey’s post-hoc test. Leaf areas were analysed by ImageJ software ver. 1.52a (National Institute of Mental Health, Bethesda, Maryland, USA) (http://imagej.nih.gov/ij).

As shown in Fig. 4, remarkable leaf area reductions were found only in the case of Phe treatment at 0.5 and 1 mM (Fig. 4).

**Figure 5:**
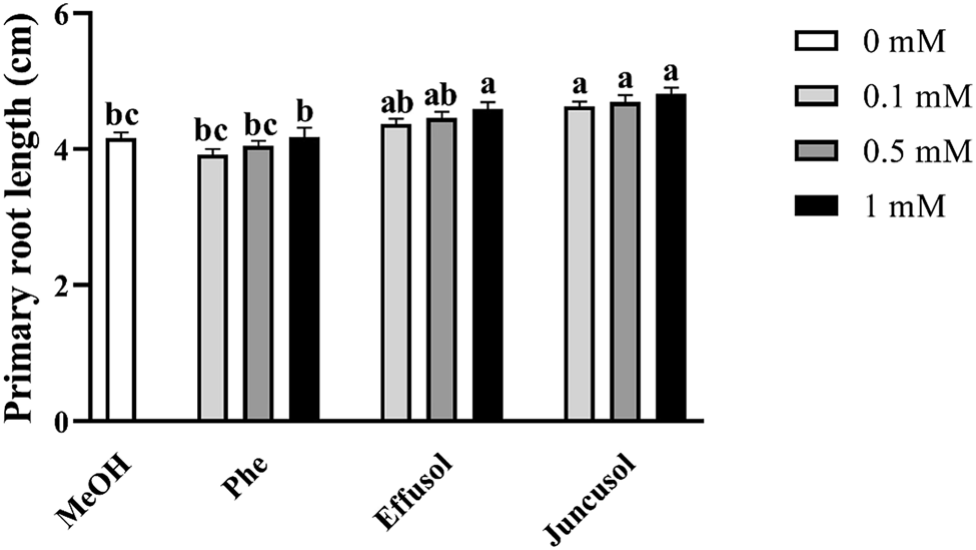
Primary root lengths of seedlings treated with Phe, effusol, and juncusol at different concentrations compared to MeOH. Data are presented as the mean ± SE (n = 30). Different letters indicate significant differences between the values of the treatments (P < 0.05) analysed by Tukey’s post-hoc test. Primary root lengths were analysed by ImageJ software ver. 1.52a (National Institute of Mental Health, Bethesda, Maryland, USA) (http://imagej.nih.gov/ij).

Primary root length was measured after 1 week of treatment with Phe, effusol and juncusol at different concentrations. The results in Fig. 5 demonstrate that in the case of effusol, only the highest concentration showed a change, while juncusol induced a longer PR length independent of the concentration compared to the methanol control and Phe at all concentrations (Fig. 5).

### Photosynthetic pigment contents

**Figure 6:**
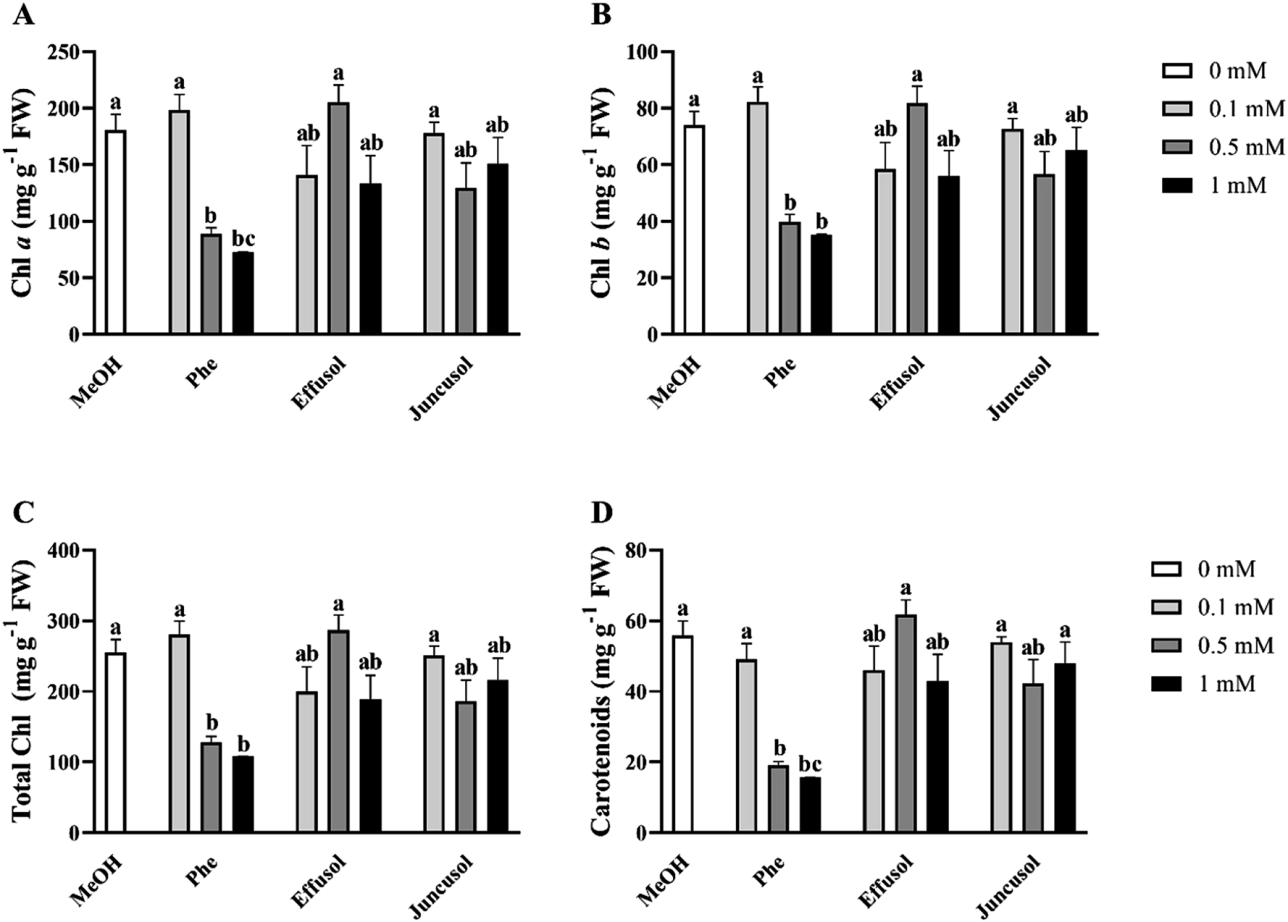
Chlorophyll and carotenoid pigment levels of seedlings treated with Phe, effusol and juncusol at different concentrations compared to MeOH. Data are presented as the mean ± SE (n = 6). Different letters indicate significant differences between the values of the treatments (P < 0.05) analysed by Tukey’s post-hoc test.

To analyse the effects of effusol and juncusol on the photosynthetic activity of *Arabidopsis* seedlings, chlorophyll-a (Chl-a), chlorophyll-b (Chl-b), carotenoids and total chlorophyll were investigated. Figure 6 shows that Phe treatment produced a drastic decrease in all photosynthetic pigments at higher concentrations, while effusol and juncusol did not cause any significant changes in photosynthetic pigment contents (Fig. 6).

### Soluble protein contents

**Figure 7:**
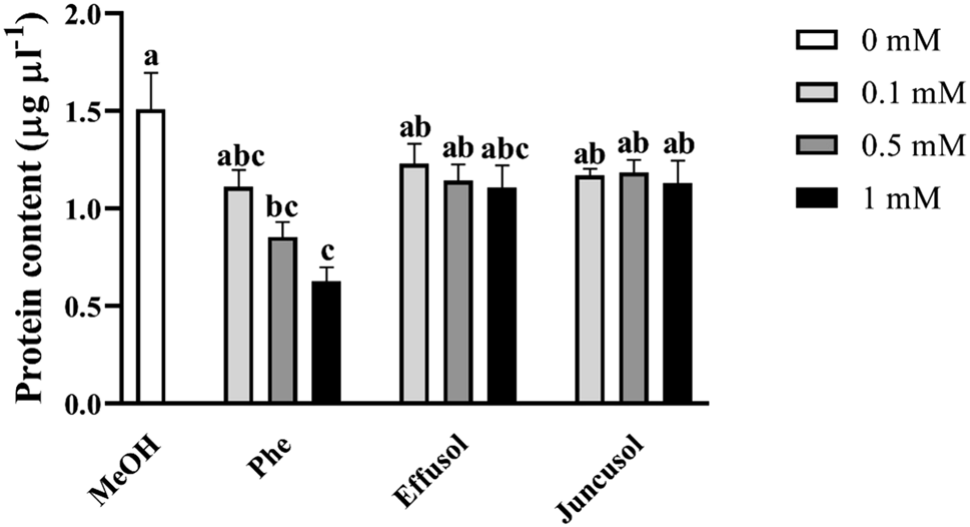
Soluble protein contents of seedlings treated with Phe, effusol and juncusol at different concentrations compared to MeOH. Data are presented as the mean ± SE (n = 6). Different letters indicate significant differences between the values of the treatments (P < 0.05) analysed by Tukey’s post-hoc test.

The soluble protein contents of *Arabidopsis* seedlings treated with Phe, effusol and juncusol were analysed. Figure 7 shows that only Phe treatment caused a drastic decrease in protein contents in a concentration-dependent manner; however, effusol- and juncusol-treated plants showed similar protein contents as the control (Fig. 7).

### Hydrogen peroxide metabolism after treatments

**Figure 8:**
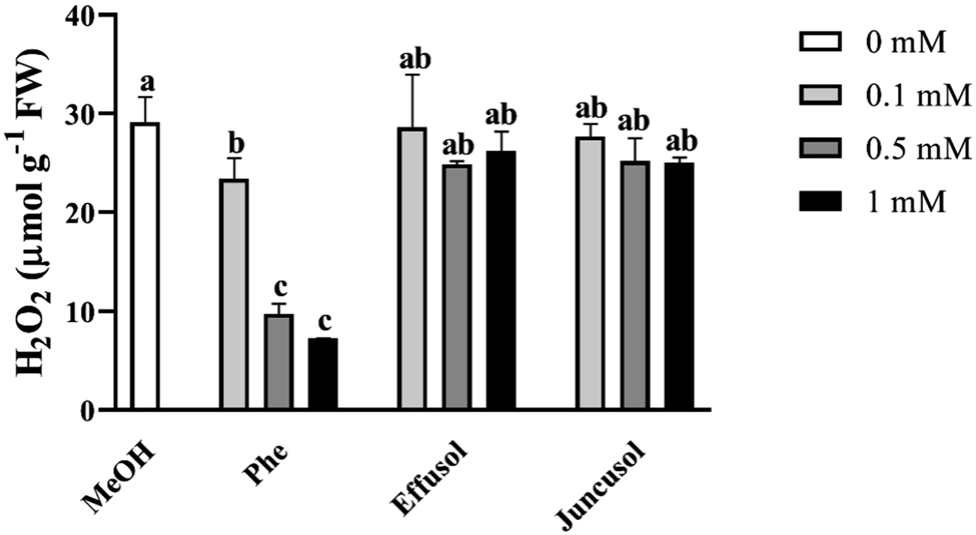
H_2_O_2_ contents of seedlings treated with Phe, effusol and juncusol at different concentrations compared to MeOH. Data are presented as the mean ± SE (n = 3). Different letters indicate significant differences between the values of the treatments (P < 0.05) analysed by Tukey’s post-hoc test.

We investigated the contents of H_2_O_2_, as this product could be synthesized during polyamine degradation in plant cells. Figure 8 demonstrates that compared to the drastic decrease in H_2_O_2_ induced by Phe, effusol and juncusol showed similar values as the control H_2_O_2_ levels, suggesting that these compounds did not induce any significant oxidative stress responses in plants.

**Figure 9:**
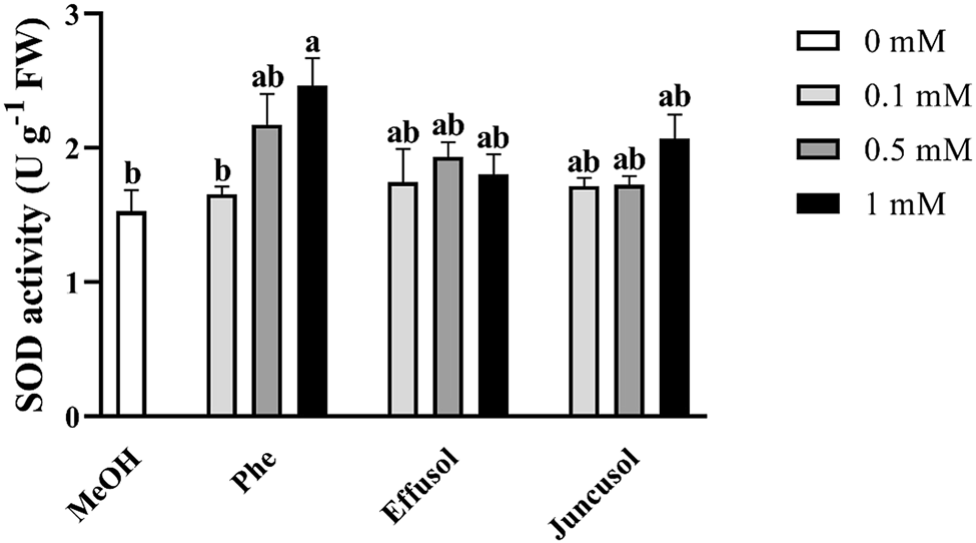
SOD activity of seedlings treated with Phe, effusol and juncusol at different concentrations compared to MeOH. Data are presented as the mean ± SE (n = 3). Different letters indicate significant differences between the values of the treatments (P < 0.05) analysed by Tukey’s post-hoc test.

We investigated the activities of SOD as it is a H_2_O_2_-producing enzyme. Only 1 mM Phe induced a significant increase in enzyme activity compared to the methanol control. There were no significant differences in SOD enzyme activity at any concentration of effusol or juncusol (Fig. 9).

**Figure 10:**
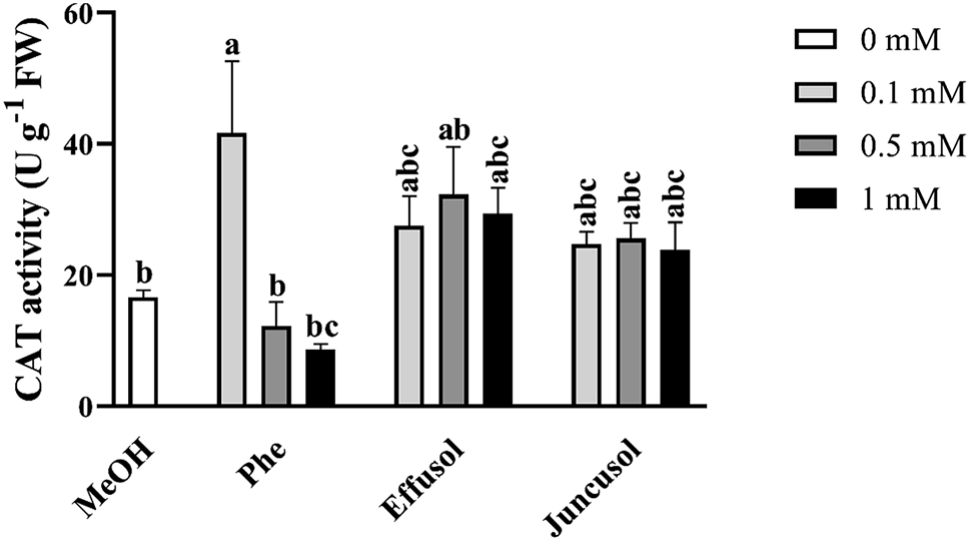
CAT activity of seedlings treated with Phe, effusol and juncusol at different concentrations compared to MeOH. Data are presented as the mean ± SE (n = 3). Different letters indicate significant differences between the values of the treatments (P < 0.05) analysed by Tukey’s post-hoc test.

To check the efficacy of the antioxidant defence mechanism, we measured the CAT and POD enzyme activities after treatment. Compared to the SOD activities, we found that only 0.1 mM Phe significantly induced CAT enzyme activity compared to the methanol control and other concentrations of Phe (Fig. 10). Moreover, effusol and juncusol did not show any significant changes in CAT activities.

**Figure 11:**
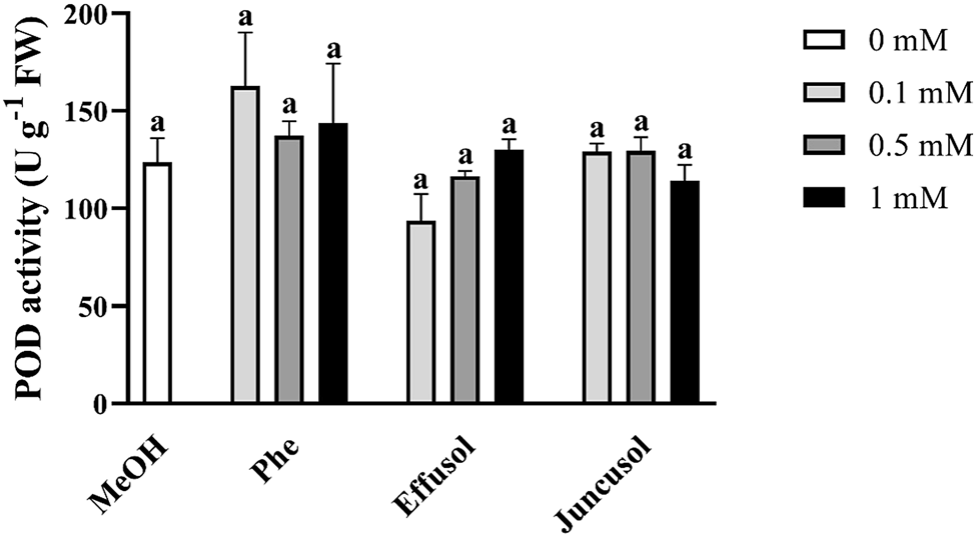
POD activity of seedlings treated with Phe, effusol and juncusol at different concentrations compared to MeOH. Data are presented as the mean ± SE (n = 3). Different letters indicate significant differences between the values of the treatments (P < 0.05) analysed by Tukey’s post-hoc test.

POD activities did not show any significant changes across all compounds and concentrations compared to the methanol control (Fig. 11).

### Free PA contents after treatments

**Figure 12:**
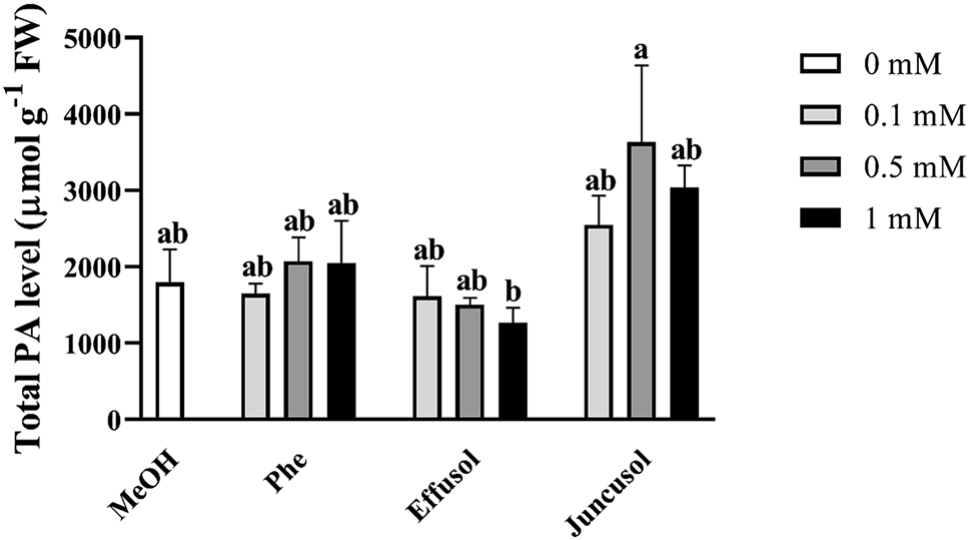
Total free PAs of seedlings treated with Phe, effusol and juncusol at different concentrations compared to MeOH. Data are presented as the mean ± SE (n = 3). Different letters indicate significant differences between the values of the treatments (P < 0.05) analysed by Tukey’s post-hoc test.

PAs are polycations that are essential to plants during their growth, development and stress responses. We investigated the effect of Phe, effusol and juncusol on the PA contents and free PA spectra in *A. thaliana* seedlings. As shown in Fig. 12, there were no significant dose-dependent effects of the compounds on the free PA levels.

**Figure 13:**
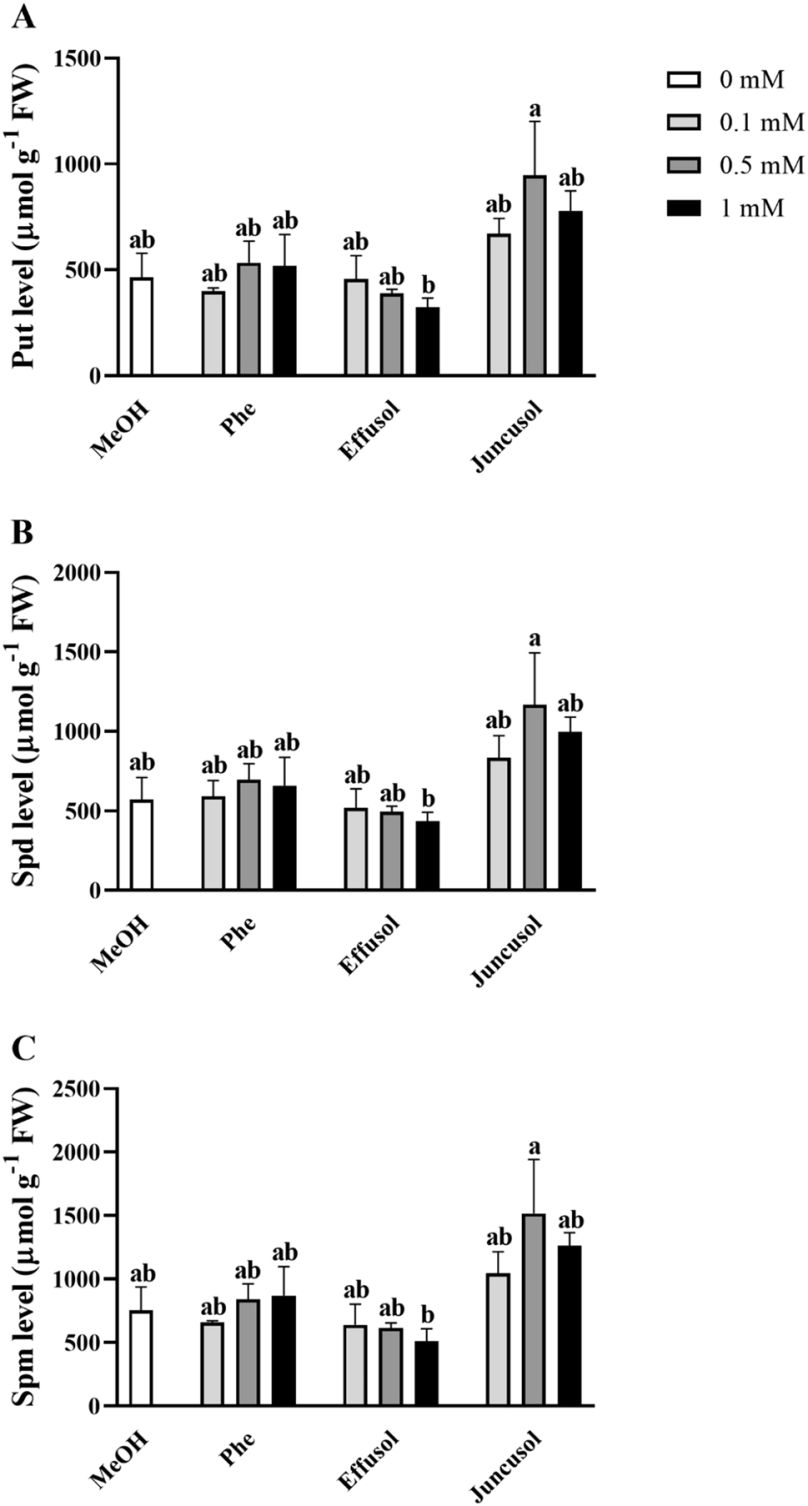
Free PAs (Put, Spd, and Spm) of seedlings treated with Phe, effusol and juncusol at different concentrations compared to MeOH. Data are presented as the mean ± SE (n = 3). Different letters indicate significant differences between the values of the treatments (P < 0.05) analysed by Tukey’s post-hoc test.

As not only the total PA pool but also the spectra of the free PAs Put, Spd, and Spm are important, their levels were determined in treated seedlings. Figure 13. shows that there were no differences in the free Put, Spd and Spm levels among the treatments.

### Ratio of higher PAs to Put

**Figure 14:**
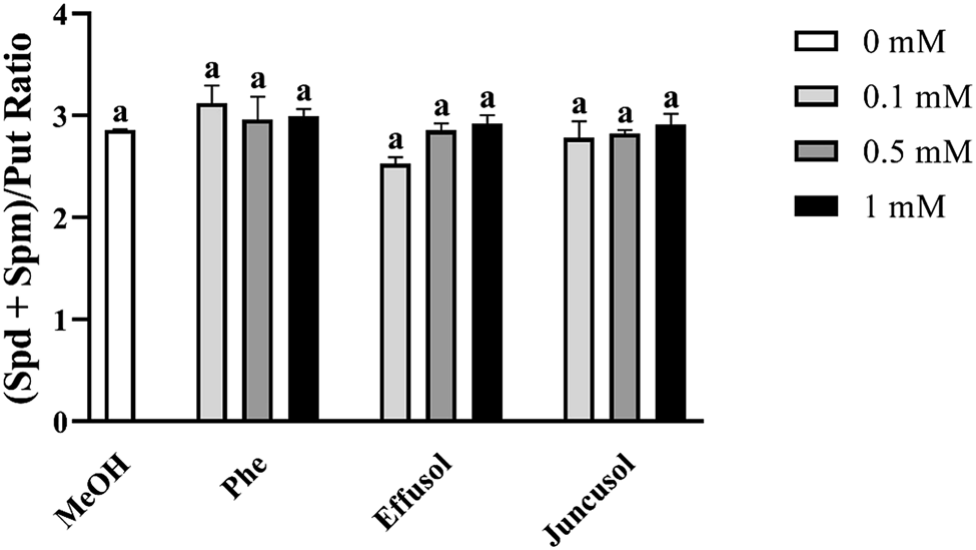
Ratio of (Spd + Spm)/Put of seedlings treated with Phe, effusol and juncusol at different concentrations compared to MeOH. Data are presented as the mean ± SE (n = 3). Different letters indicate significant differences between the values of the treatments (P < 0.05) analysed by Tukey’s post-hoc test.

The ratio of higher PAs (Spd and Spm) to Put is also crucial for optimal PA metabolism in plants. Accumulating more Put than higher PAs could induce cell death mechanisms in plants. The ratio of higher PAs to Put did not change after treatments (Fig. 14).

### Polyamine catabolism after treatment

**Figure 15:**
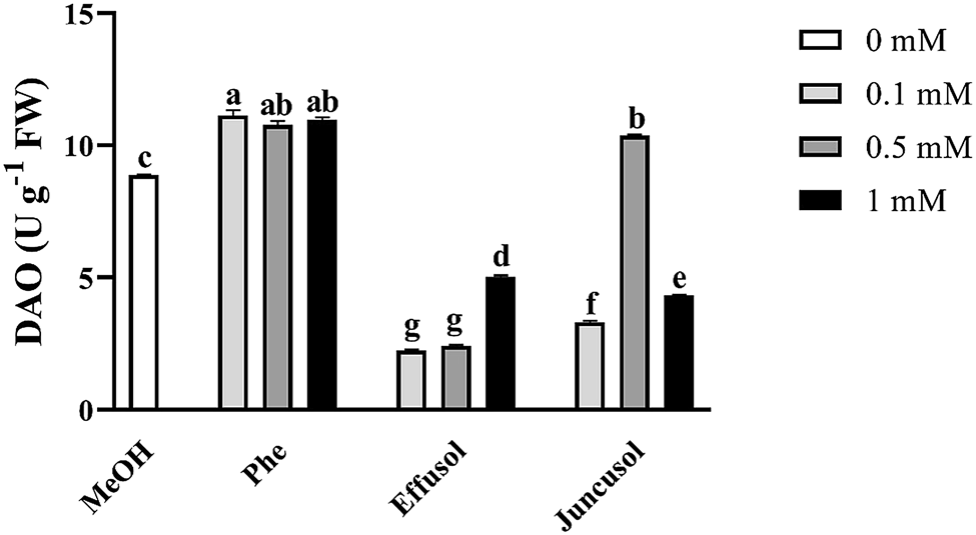
DAO enzyme activities of seedlings treated with Phe, effusol and juncusol at different concentrations compared to MeOH. Data are presented as the mean ± SE (n = 3). Different letters indicate significant differences between the values of the treatments (P < 0.05) analysed by Tukey’s post-hoc test.

**Figure 16:**
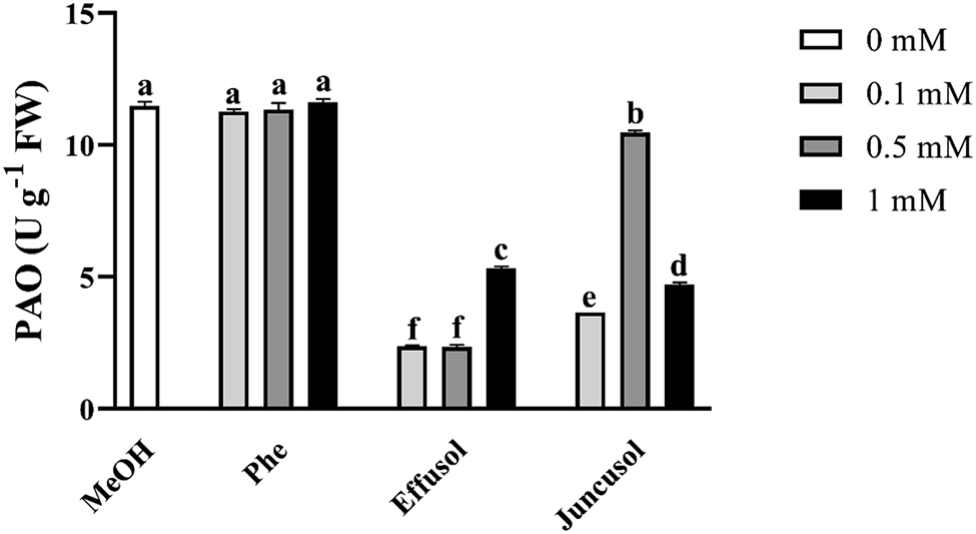
PAO enzyme activities of seedlings treated with Phe, effusol and juncusol at different concentrations compared to MeOH. Data are presented as the mean ± SE (n = 3). Different letters indicate significant differences between the values of the treatments (P < 0.05) analysed by Tukey’s post-hoc test.

PA-degrading enzyme activity induced by Phe, effusol and juncusol was evaluated in *A. thaliana* seedlings. PA catabolism was determined based on the enzyme activities of Put degrading DAO and higher PAs degrading PAO after 1 week of treatment with three compounds at different concentrations. As we expected, Phe treatment elicited the most intensive responses at all applied concentrations for DAO, while there were no changes in PAO activity with the different concentrations of Phe regarding to the MeOH control. In the case of effusol and juncusol treatment, all concentrations used caused a large decrease in both enzyme activities, except for juncusol at 0.5 mM, where DAO increased (Fig. 15). PAO activity diminished with all effusol and juncusol concentrations used (Fig. 16).

## Discussion

It has been widely reported that Phe can induce plant growth retardation by reducing biomass and photosynthesis^39–41^. Despite many studies focused on Phe, phenanthrene-like compounds have rarely been investigated in higher plants. In this study, we investigated the concentration-dependent effects of effusol and juncusol from Juncaceae on *Arabidopsis thaliana* as a model plant.

Similar to other studies^40^, the toxic effects of Phe on seedling growth parameters were demonstrated in our study, while for effusol and juncusol treatment, a growth promotion effect via increased biomass, enhanced leaf areas, and increased primary root lengths was found, mainly for juncusol-treated seedlings (Figs. 3, 4, 5). In contrast to our results, Della Greca et al.^42^ reported that aglycons isolated from *J. effuses* showed a strong inhibitory activity on the unicellular green alga *S. capricornutum*; however, the glucosides were inactive or had a slight stimulating effect on algal growth.

Phe was reported to cause a significant inhibition of photosynthesis in plants^40^. Our findings show that Phe was inhibitory when applied at higher concentrations, but effusol and juncusol, regardless of the concentration, did not cause any significant changes, suggesting that these two compounds could induce growth promotion without any effect on photosynthetic pigments (Fig. 6).

By comparing the biochemical parameters of seedlings, we analysed the total soluble protein contents. Based on our results, significant reductions in protein levels occurred only in the case of seedlings treated with 0.5 and 1 mM concentrations of Phe, which could be associated with reduced growth. Effusol and juncusol did not show any significant changes compared to the methanol treatment (Fig. 7).

H_2_O_2_ is one of the most important forms of reactive oxygen species and plays an important role in developmental processes and stress responses. Phe induced a hypersensitive response in *Arabidopsis thaliana,* indicating that Phe could be able to induce oxidative stress in plants^39^. It has been reported that juncusol, effusol and other related compounds are cytotoxic to animals^43^, also suggesting their relation to oxidative stress. It is important to note that only Phe induced decreased H_2_O_2_ levels related to toxic effects, while effusol and juncusol did not display any alterations in H_2_O_2_ contents, suggesting an effective antioxidant mechanism (Fig. 8). To provide evidence for this hypothesis, we investigated the enzyme activities of SOD, CAT and POD as the most important stress marker enzymes involved in H_2_O_2_ metabolism. Phe induced a decrease in the H_2_O_2_ content simultaneously with increased SOD and reduced CAT activities, suggesting that H_2_O_2_ could be converted to more reactive hydroxyl anions due to the Fenton-Haber Weiss reaction to oxidize Phe, as shown for soil remediation technologies^44,45^. Effusol and juncusol treatments induced similar enzyme activities compared to methanol treatment, suggesting that these compounds did not cause any oxidative stress in plants (Figs. 9, 10).

Polyamines are essential polycationic plant growth regulators with an important role in plant growth and development and in stress responses^29^. PA metabolism has also been found to be associated with Phe in the aquatic water plant *Riccia fluitans*^27^. PA metabolism could be affected by the balance between the biosynthetic production and degradation of PAs. In this study, free PA contents did not significantly change regardless of compounds or their concentrations (Figs. 12, 13, 14). However, in the case of degrading enzyme activities (DAO and PAO), Phe induced higher DAO activities, but not PAO activities, at all concentrations compared to methanol treatment. Interestingly, remarkable differences could be seen between the compounds and their concentrations. Effusol was the most effective at reducing PA degradation, while juncusol at 0.5 mM induced a slight increase in enzyme activity in the case of DAO, in contrast to PAO. These results do not agree with the contents of free PAs, suggesting that other types of PAs (membrane-bound or conjugated forms) could be involved in the background effects of these compounds. We hypothesize that effusol and juncusol contribute to fine-tuning PA metabolism.

## Concluding remarks

Our results revealed that exogenous application of two phenanthrenoid compounds from *Juncus compressus*, effusol and juncusol, promoted the growth of *Arabidopsis thaliana* seedlings. Compared to the growth retardation induced by Phe, the effects of these two compounds induced higher biomass, thus maintaining an optimal antioxidant defence system that was associated with reduced polyamine degradation. It is important to note that juncusol was more reactive at the lower concentrations used in this study, while the concentration of effusol could be elevated to induce biostimulant effects, but the mechanism of biomass stimulation requires further investigation. In the future, these compounds could be good candidates for new biopesticide or biostimulant plant growth regulators in a concentration-dependent manner.
